# Interleukin-10 Inhibits Lipopolysaccharide Induced miR-155 Precursor Stability and Maturation

**DOI:** 10.1371/journal.pone.0071336

**Published:** 2013-08-12

**Authors:** Sylvia T. Cheung, Eva Y. So, David Chang, Andrew Ming-Lum, Alice L-F. Mui

**Affiliations:** 1 Immunity and Infection Research Centre, Vancouver Coastal Health Research Institute, Vancouver, British Columbia, Canada; 2 Department of Surgery, University of British Columbia, Vancouver, British Columbia, Canada; 3 Department of Biochemistry and Molecular Biology, University of British Columbia, Vancouver, British Columbia, Canada; International Center for Genetic Engineering and Biotechnology, India

## Abstract

The anti-inflammatory cytokine interleukin-10 (IL-10) is essential for attenuating the inflammatory response, which includes reducing the expression of pro-inflammatory microRNA-155 (miR-155) in lipopolysaccharide (LPS) activated macrophages. miR-155 enhances the expression of pro-inflammatory cytokines such as TNFα and suppresses expression of anti-inflammatory molecules such as SOCS1. Therefore, we examined the mechanism by which IL-10 inhibits miR-155. We found that IL-10 treatment did not affect the transcription of the miR-155 host gene nor the nuclear export of pre-miR-155, but rather destabilized both pri-miR-155 and pre-miR-155 transcripts, as well as interfered with the final maturation of miR-155. This inhibitory effect of IL-10 on miR-155 expression involved the contribution of both the STAT3 transcription factor and the phosphoinositol phosphatase SHIP1. This is the first report showing evidence that IL-10 regulates miRNA expression post-transcriptionally.

## Introduction

Macrophage activation in response to pathogens is an important part of host defense. When the bacterial cell wall product lipopolysaccharide (LPS) binds to the Toll-like receptor 4 (TLR4) on the macrophage, a cascade of signalling pathways is triggered leading to the production of pro-inflammatory cytokines and other inflammatory mediators [1]. However, this inflammatory response must be appropriately terminated to avoid pathological consequences [2]–[4]. The anti-inflammatory cytokine interleukin-10 (IL-10) is a key inhibitor in both mice and men. IL-10 deficient mice develop spontaneous colitis and show exaggerated inflammatory responses to infection [5], [6], while deficiencies in IL-10 production or mutations in the IL-10 receptor result in inflammatory diseases in men [7]–[10]. The importance of IL-10 in regulating immune cell function is further illustrated by the fact that many tumour cells and intracellular pathogens produce or elicit production of IL-10 to enhance survival [11].

IL-10 binding to its receptor (IL-10R) leads to activation of receptor associated Jak1 and Tyk2 tyrosine kinases, and subsequent activation of the signalling transducer and activator of transcription 3 (STAT3) pathway [12]–[14]. In addition to the STAT3 pathway, we have recently shown that IL-10 also signals through the SH2 domain containing inositol 5′phosphatase 1 (SHIP1) [15] (Ming-Lum *et al*., submitted). SHIP1 negatively regulates phosphoinositide 3-kinase (PI3K) signalling by hydrolyzing the PI3K product, phosphatidylinositol-3,4,5-P_3_ (PIP_3_) [16], [17]. Degradation of PIP_3_ inhibits the function of PIP_3_-dependent signalling proteins such as the protein kinase AKT [18]. We found that IL-10 inhibited LPS activation of AKT through SHIP1 (Ming-Lum *et al*., submitted).

microRNAs (miRNAs) have been recognized as a new class of regulatory molecules in eukaryotic cells. miRNAs are small non-coding RNAs that regulate target mRNA translation and stability in the cytoplasm. Long primary miRNA transcripts (pri-miRNAs) are transcribed by RNA polymerase II [19], processed by Drosha [20] to form precursor (pre-) miRNAs, and followed by nuclear export aided by Exportin-5 [21]. The pre-miRNAs are further processed by the RNase Dicer, and the mature miRNAs are loaded onto the RNA-induced silencing complex (RISC). The specific binding of miRNAs to the 3′untranslated region (UTR) of target mRNAs can lead to either translational repression or mRNA degradation [22]. Up to 52% of innate immune genes have conserved miRNAs’ target sites, indicating major roles of miRNAs in immune regulation [23]. When the gene encoding for Dicer was deleted in macrophages, expression of LPS-induced cytokines such as IL-1β and IL-10 were enhanced, indicating that miRNAs are important in LPS-induced macrophage activation [24].

Of the known miRNAs that can be induced by LPS in macrophages [25], miR-155 has been one of the most extensively studied. miR-155 is processed from an exon of a noncoding RNA transcribed from the B cell integration cluster (BIC), a gene which is strongly conserved among human, mouse and chicken [26]. While unrestricted expression of miR-155 has been associated with cancer [27]–[29], miR-155 knockout mice displayed aberrant immune functions including defective B and T cell immunity and abnormal function of antigen-presenting cells [30]. On the cellular level, miR-155 expression is strongly induced by different TLR ligands including LPS [31]. In-depth animal studies and experiments using luciferase based reporter genes or anti-miR antagomir showed that miR-155 targets at least 20 genes in immune cells, including SHIP1 [32], [33] and SOCS1 [34], both of which are negative regulators of macrophage activation. Consistent with the pro-inflammatory properties of miR-155, TNFα translation is enhanced by the presence of miR-155 via increasing mRNA stability [25], [35], [36]. Due to its wide ranging effects on immune cell functions, expression of miR-155 has to been tightly controlled. A recent study suggested that IL-10 could inhibit LPS-induced miR-155 expression in macrophages in a STAT3-dependent manner [37].

In this study, we hypothesized that in addition to the STAT3 pathway, the phosphoinositol phosphatase SHIP1 pathway may play a role in IL-10 inhibition of miR-155. We found that IL-10 indeed utilized both STAT3 and SHIP1 to inhibit LPS-induced miR-155 expression. We also found that IL-10 did not alter the transcription of pri-miR-155 or the nuclear export of pre-miR-155; rather, IL-10 reduced the stability of pri-miR-155 and pre-miR-155 transcripts and inhibited the maturation of miR-155.

## Materials and Methods

### Ethics Statement

Cells were harvested from mice for some of the studies. This study was carried out in strict accordance with the recommendations and guidelines of University of British Columbia Animal Care Committee which approved protocol #A11-0218 for our study.

### Cells and Reagents

RAW264.7 cells were obtained from American Type Culture Collection and cultured in Dulbecco’s Modified Eagle Medium (DMEM) supplemented with 9% (v/v) fetal calf serum (FCS) (Thermo Fisher Scientific, Nepean, ON). Generation of the doxycycline (Dox) inducible Scrambled siRNA and SHIP1 knockdown cell lines are as described previously [15]. To generate the AKT-ER stable cell line, RAW264.7 cells were transduced (University of British Columbia Biosafety certificate # B12-0010) with an AKT-ER construct as described in the “Plasmids and Lentivirus” section below. AKT-ER expressing cells were selected by growth in 5 µg/ml blasticidin. Primary peritoneal macrophages (perimacs) were isolated from 6 to 8 weeks old male and female Balb/c wild-type (WT) or SHIP1 knockout (SHIP1 KO) mice (Dr. Gerald Krystal, BC Cancer Research Centre, Vancouver, BC) by peritoneal lavage with 3 ml of sterile Phosphate Buffered Saline (PBS, Thermo Fisher Scientific, Nepean, ON). Perimacs were collected and transferred to Iscove’s Modified Dulbecco’s Medium (Thermo Fisher Scientific, Nepean, ON) supplemented with 10% (v/v) FCS, 10 µM β-mercaptoethanol, 150 µM monothioglycolate and 1 mM L-glutamine. All cells were maintained in a 37°C, 5% CO_2_, 95% humidity incubator.

Antibodies used include anti-SHIP1 (P1C1) mouse antibody (Santa Cruz Biotechnology, Santa Barbara, CA) and anti-STAT3 mouse antibody (BD Transduction lab, Mississauga, ON). AQX-MN100 (Aquinox Pharmaceuticals, Vancouver, BC) was dissolved in ethanol, and STA-21 (Cedarlane Laboratories, Burlington, ON) was dissolved in dimethylsulfoxide (DMSO).

### Plasmids and Lentivirus Production

Luciferase reporter plasmids containing the BIC promoter were obtained from Dr. Eric Flemington (Tulane University, New Orleans, LA) [7]. The mouse IkBζ promoter luciferase reporter was constructed into the pGL3-basic plasmid (Promega, Madison, WI) between the SacI and NheI sites. This construct contained the IkBζ promoter fragment (−400 to +1) generated by reverse transcription and PCR amplification from total mouse RNA. The c-fos promoter reporter is previously described [38].

A plasmid construct containing a modified form of human AKT was kindly provided by Dr. Megan Levings (University of British Columbia, Vancouver, BC) [39]. This AKT construct lacks the PH domain but has a src myristoylation signal sequence at the amino terminal end and the steroid binding domain of the estrogen receptor (ER) and a hemagglutinin tag at the carboxyl terminal end [40]. The AKT-ER sequence was sub-cloned into the pENTR-1A vector (Invitrogen, Mississauga, ON) and recombined into a modified lentiviral vector, pTRIPZ. VSV-pseudotyped second-generation lentiviruses were produced by transient 3-plasmid co-transfection into HEK293T cells and concentrated by ultracentrifugation.

### Cell Stimulations

RAW264.7 cells were seeded at 1.5×10^6^ cells per well on 6-well tissue culture plates or 3×10^5^ cells per well on 24-well tissue culture plates 1 day prior to stimulation. The SHIP1 siRNA-transduced cell lines were left untreated or treated with 2 µg/ml Dox for 24 hours prior to cell seeding (a total of 48 hours treatment before stimulation) to induce knockdown of SHIP1. The AKT-ER transduced cells were pretreated with 150 nM 4-hydroxytamoxifin (4-HT) for 20 minutes prior to stimulation. Perimacs were seeded at 3×10^6^ cells per well in 6-well tissue culture plate and let to adhere overnight before stimulation. For the STA-21 experiments, 30 µM STA-21 or DMSO control was added to the cells 1 hour prior to stimulation. Cells were stimulated with 1–10 ng/ml LPS (*E. coli* Serotype 0111:B4) with or without the indicated concentrations of IL-10.

### Luciferase Reporter Analysis

RAW264.7 cells were seeded at 2×10^5^ cells per well on 24-well tissue culture plates 4 hours before transfection. Each promoter reporter plasmid was co-transfected with phRL-TK using the XtremeGene HP transfection reagent (Roche Diagnostics, Laval, QC) according to manufacturer’s instruction. Cells were rested for 24 hours prior to stimulation with LPS +/− IL-10. Cells were then lysed in 200 µl of 1X Passive Lysis Buffer (Promega, Madison, WI) and luciferase activities were measured using the Dual-Luciferase Reporter Assay System (Promega, Madison, WI). The typical transfection efficiency in RAW264.7 macrophages was about 20%.

### Fractionation of Nuclear and Cytoplasmic RNA

After stimulation, cells were rinsed with PBS and lysed in lysis buffer containing 10 mM Tris-HCl pH7.4, 150 mM NaCl, 1.5 mM MgCl_2_ and 0.65% Nonidet P-40, supplemented with 100 unit/ml RNase inhibitor (Roche Diagnostics, Laval, QC) for 30 minutes at 4°C. Nuclei were pelleted by centrifugation and the supernatant (cytoplasmic fraction) was transferred to a new tube. Both cytoplasmic and nuclear fractions were then prepared in Trizol reagent (Invitrogen, Burlington, ON) for RNA extraction.

### Immunoblot Analysis

Cells were lysed with lysis buffer containing 50 mM HEPES, 2 mM EDTA, 1 mM NaVO_4,_ 100 mM NaF, 50 mM NaPP_i_ and 1% Nonidet P-40, supplemented with Complete Protease Inhibitor Cocktail (Roche Diagnostics, Laval, QC). Lysates were incubated at 4°C for 30 minutes and clarified by centrifuging for 20 minutes at 12,000 g. Proteins were then separated on a 7.5% SDS-PAGE and transferred onto PVDF membrane (Millipore, Etobicoke, ON). The membrane was blocked, probed with the indicated primary antibodies overnight, washed, developed with the Alexa Fluor® 660 anti-mouse IgG antibody (Invitrogen, Burlington, ON) and imaged using a LICOR Odyssey Imager. Band intensities were quantified using the Quantity One Software (Biorad, Missisauga, ON).

### RNA Extraction and Real Time PCR

Total RNA was extracted using Trizol reagent (Invitrogen, Burlington, ON) according to manufacturer’s instructions. About 2–5 µg of RNA was treated with DNAseI (Roche Diagnostics, Laval, QC) according to product manual. For miRNA expression analysis, 20 ng of RNA was used as the starting material in miRNA TaqMan assays (Applied Biosystems, Burlington, ON) according to manufacturer’s instructions. For mRNA expression analysis, 120 ng of RNA was used in the Transcriptor First Strand cDNA synthesis kit (Roche Diagnostics, Laval, QC), and 0.1 µl to 0.2 µl of cDNA generated was analyzed by SYBR Green-based real time PCR (real time-PCR) (Roche Diagnostics, Laval, QC) using 300 nM of gene-specific primers. The following primers were used: **pri-miR-155**: forward, 5′-GACACAAGGCCTGTTACTAGCAC-3′, reverse, 5′-GTCTGACATCTACGTTCATCCAGC-3′; **pre-miR-155**: forward, 5′-GCTAATTGTGATAGGGGTTTTGG-3′, reverse, 5′-GTTAATGCTAACAGGTAGGAGTC-3′; **TNFα**: forward, 5`-TCTTCTCATTCCTGCTTGTGG-3′, reverse, 5`-GGTCTGGGCCATAGAACTGA-3′; **18S rRNA**: forward, 5′-CAAGACGGACCAGAGCGAAA-3′, reverse, 5′-GGCGGGTCATGGGAATAAC-3′; **GAPDH**: forward, 5′-AATGTGTCCGTCGTGGATCT-3′, reverse, 5′-GCTTCACCACCTTCTTGATGT-3′. Expression of miRNA and mRNA was measured with the 7300 RT-PCR system (Applied Biosystems, Burlington, ON), and the comparative Ct method was used to quantify miRNA or mRNA levels using snoRNA202 or GAPDH as the normalization control. In the CHX studies, 18S rRNA was used as normalization control instead of GAPDH.

### Statistical Analysis

All statistical analysis was performed using GraphPad Prism 6 software.

## Results

**Figure 1:**
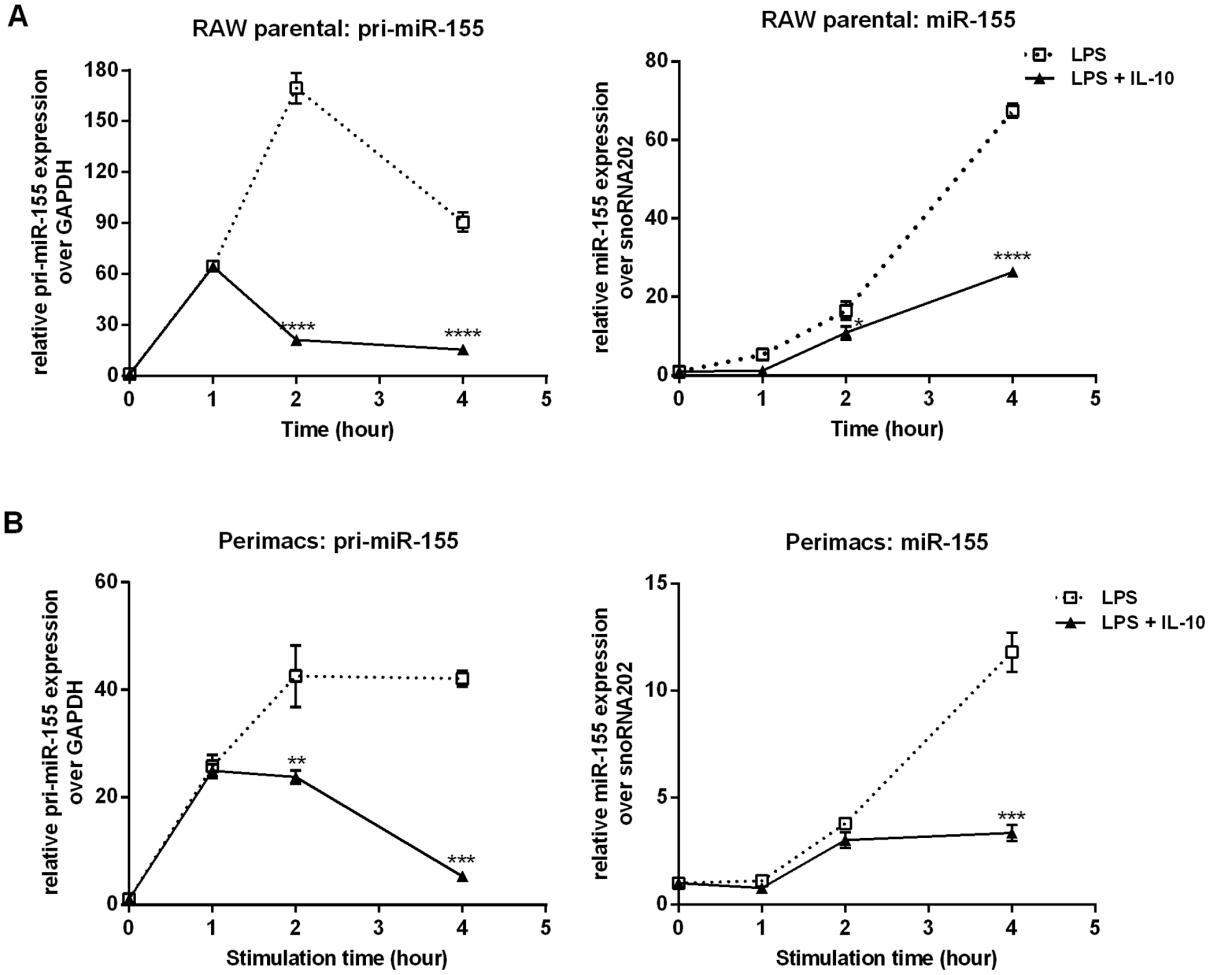
IL-10 inhibits LPS induction of pri-miR-155 and miR-155 expression in macrophages. (A) RAW264.7 parental cells or (B) perimacs were stimulated with LPS +/− IL-10 for the indicated times prior to total RNA extraction. Expression levels of pri-miR-155 and miR-155 were determined by real time PCR and plotted relative to unstimulated samples. Statistical significance between LPS and LPS+IL-10 treatment was calculated by a two-way ANOVA test with a 95% confidence (*p<0.05, **p<0.01, ***p<0.001). Results were observed in at least three independent experiments.

We first examined the kinetics of IL-10 inhibition of pri-miR155 and mature miR155. As shown in Figure 1A, LPS-induced expression of pri-miR-155 was detected as early as 1 hour in the RAW264.7 macrophage cell line, peaked at 2 hours and declined after that. The level of mature miR-155, on the other hand, was barely detectable at 1 hour and continued to increase over the course of 4 hours. Addition of IL-10 inhibited expression of pri-miR-155, but inhibition was only observed after 1 hour and was statistically significant after 2 hours and later. IL-10 inhibition of miR-155 was also delayed, with inhibition being statistically significant only at 4 hours. Similar kinetics was observed in peritoneal macrophages freshly isolated from mice (Figure 1B). The inhibitory effect of IL-10 on pri-miR-155 and miR-155 levels are similar to that reported previously [37].

### LPS and IL-10 do not Regulate miR-155 at the Level of Transcription

**Figure 2:**
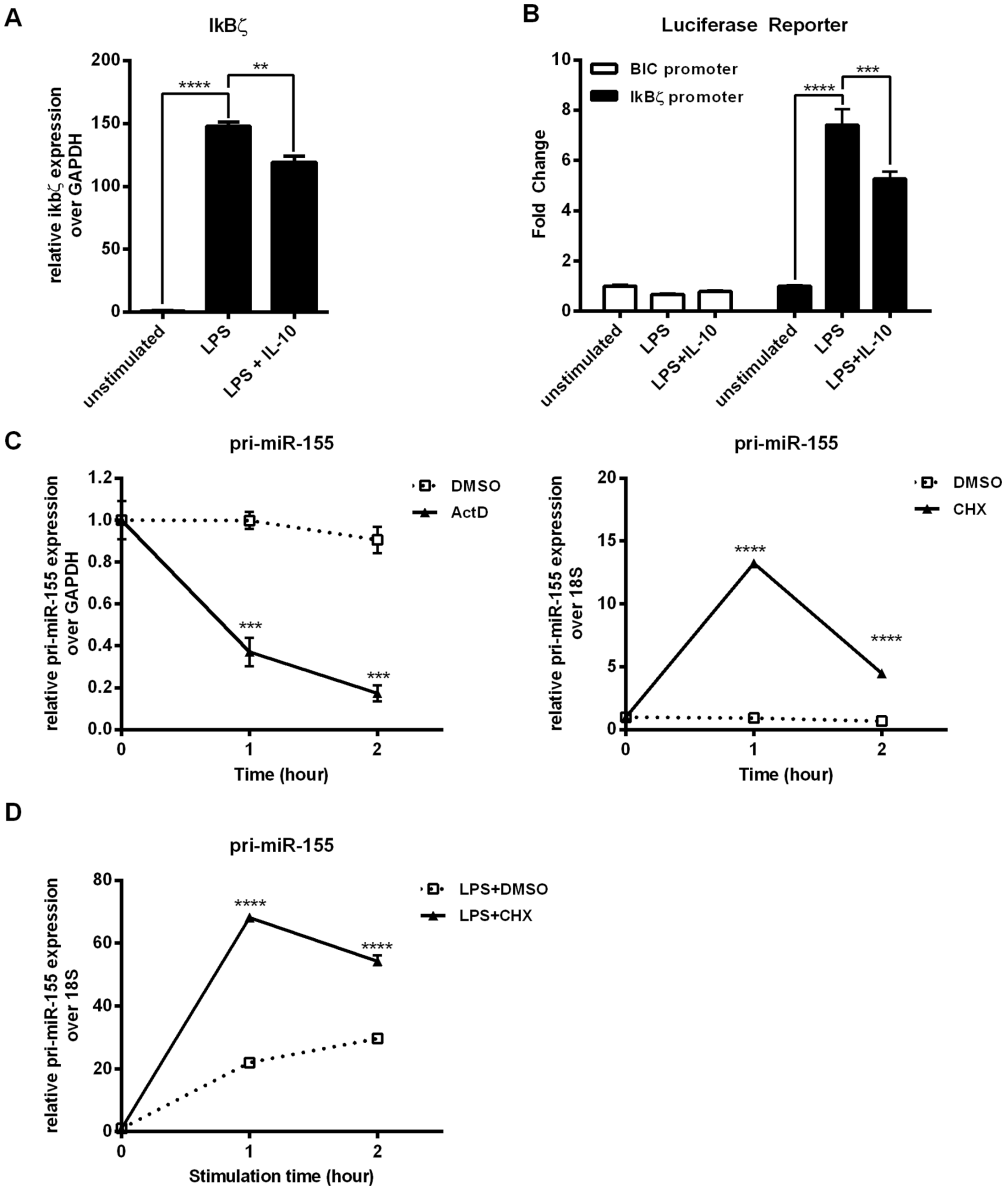
IL-10 does not regulate miR-155 expression at the transcription level. (A) RAW264.7 parental cells were stimulated with LPS +/− IL-10 for 1 hour prior to total RNA extraction. Expression levels of IkBζ were determined by real time PCR and plotted relative to unstimulated samples. Statistical significance between treatment was calculated by an unpaired two-tailed student’s t-test with a 95% confidence (**p<0.01, ****p<0.0001). (B) RAW264.7 cells were transfected with TK-Renilla and BIC promoter reporter or IkBζ promoter reporter. After 24 hours rest, cells were stimulated with LPS +/− IL-10 for 2 hours. Reporter activity was normalized to the TK-Renilla and plotted as fold change relative to the unstimulated sample. (C – D) RAW264.7 cells were treated with (C) ActD, CHX or DMSO, or (D) LPS+DMSO or CHX for the indicated time prior to RNA extraction and determination of pri-miR-155 level by real time PCR. Statistical significance between DMSO treatment and drug treatment was calculated by a two-way ANOVA test with a 95% confidence (****p<0.0001). Results were observed in at least two independent experiments.

Several check points are in place to regulate the level of particular miRNAs in cells: transcription of pri-miRNA, Drosha-mediated generation of pre-miRNA, export of pre-miRNA and finally Dicer-mediated maturation of miRNA [41]. The kinetics of miR-155 expression in response to LPS +/− IL-10 (Figure 1A–B) suggested that the regulation of pri-miR-155 and mature miR-155 differs. We first examined the potential effect of LPS and IL-10 on the transcription of pri-miR-155 by using a luciferase reporter construct controlled by the BIC promoter (the host gene of miR-155) [42]. A reporter harbouring the promoter of IkBζ acted as the control for our reporter assays. IkBζ is a known LPS response gene [43]. As shown by real time PCR, we found that IL-10 inhibited LPS-induced IkBζ mRNA expression in RAW264.7 cells (Figure 2A). The IkBζ promoter reporter showed similar LPS induction and IL-10 inhibition pattern (Figure 2B). In contrast, we found that LPS did not induce BIC promoter activity compared to the unstimulated control (Figure 2B). Similarly, addition of IL-10 did not affect the activity of the BIC promoter either. The data were surprising since pri-miR-155, the primary transcript from the BIC gene, increased with LPS stimulation and decreased with IL-10 treatment (Figure 1A). Also, the unresponsiveness of the BIC reporter to stimuli differs from McCoy *et al.*’s finding that LPS stimulated, while IL-10 inhibited, BIC reporter activity [37]. We assessed whether the difference between our and McCoy *et al.’*s BIC reporter results might be due to cell stimulation time, transfection reagent used, and/or transfection times (Figure S1). We consistently observed no change in the BIC reporter activity upon LPS and IL-10 treatment.

The lack of responsiveness of the BIC reporter to LPS and IL-10 was unexpected since LPS clearly increased the level of pri-miR-155 in cells (Figure 1). However, steady state transcript level of a gene is not a sole result of increased transcription; it can also be due to decreased transcript degradation. To examine the possibility that the pri-miR-155 is constitutively transcribed and undergoes regulated degradation, we looked at the effect of the transcription inhibitor actinomycin D (ActD) or translation inhibitor cycloheximide (CHX) on pri-miR-155 levels. In the experiments using CHX, we used 18S rRNA as the normalization control, instead of GAPDH, because GAPDH expression level was sensitive to CHX treatment while 18S rRNA expression level was not (Figure S2). We treated resting RAW264.7 with ActD and found that steady state pri-miR-155 level dropped more than 2-fold by 1 hour and was almost undetectable at 2 hours (Figure 2C, left panel). This suggests that pri-miR-155 is constitutively transcribed even in unstimulated RAW264.7 cells. On the other hand, CHX treatment increased pri-miR-155 levels by 6-fold in 1 hour suggesting *de novo* translation of short-lived decay factors contributes to keeping pri-miR-155 levels down in unstimulated cells (Figure 2C, right panel). CHX treatment also enhanced LPS-induced pri-miR-155 expression (Figure 2D).

### LPS and IL-10 do not Regulate Nuclear Export of Pre-miR-155

**Figure 3:**
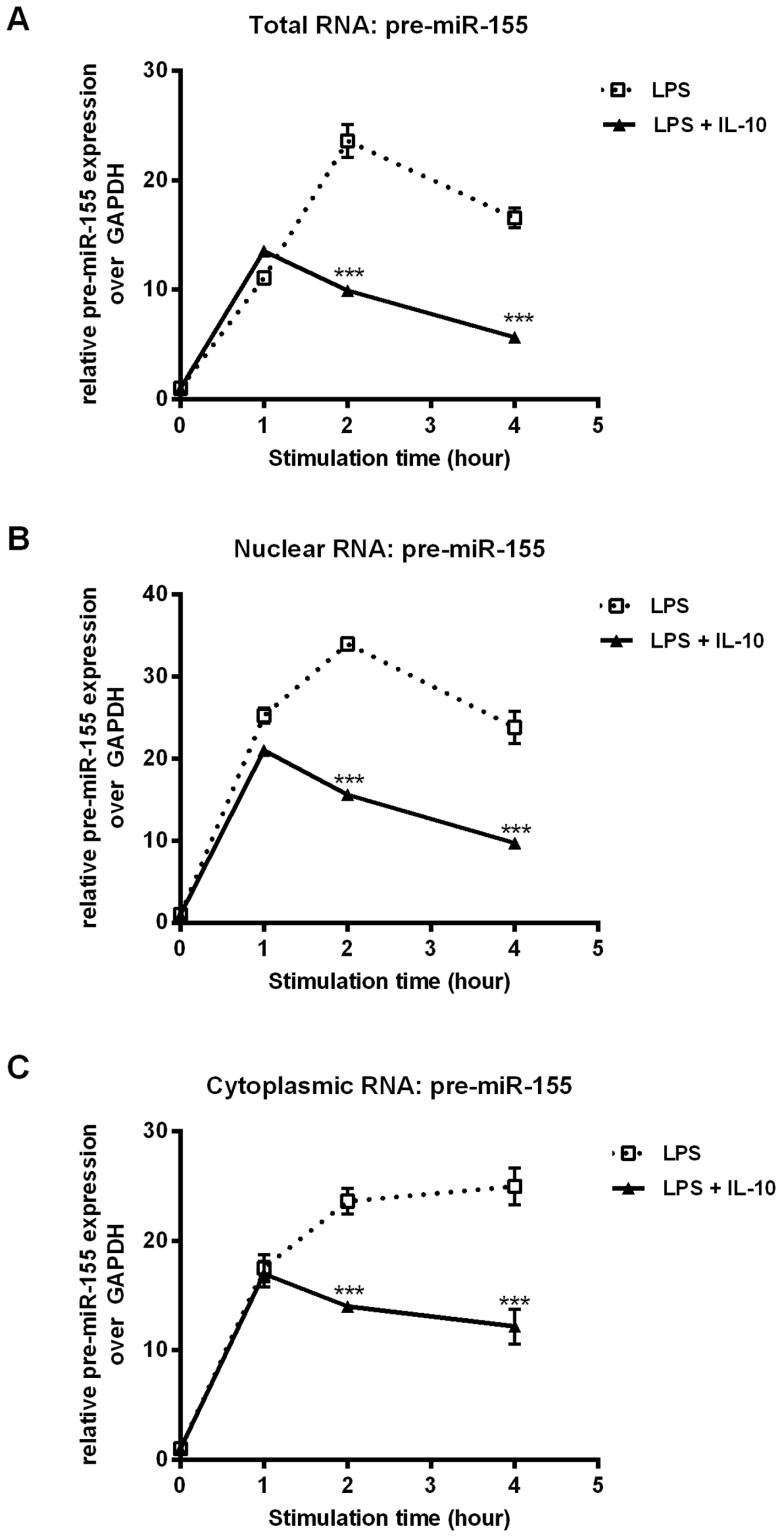
IL-10 does not affect the export of pre-miR-155 from the nucleus to the cytoplasm. RAW264.7 cells were stimulated with LPS +/− IL-10 for the indicated times prior to fractionation of nuclei and cytoplasm. Levels of pre-miR-155 in (A) total, (B) nuclear and (C) cytoplasmic fractions were determined by real time PCR. Statistical significance between LPS and LPS+IL-10 treatment was calculated by a two-way ANOVA test with a 95% confidence (*p<0.05, **p<0.01, ***p<0.001). Results were observed in at least three independent experiments.

Another miRNA regulation check point occurs at the export of pre-miRNAs from the nucleus to the cytoplasm. The delayed expression of mature miR-155 relative to pri-miR-155 and pre-miR-155 might be due to delayed export of pre-miR-155 into the cytoplasm for processing by Dicer. To investigate the possible effect of LPS and IL-10 on the nuclear export of pre-miR-155, we stimulated RAW 264.7 cells with LPS +/− IL-10 and fractionated the cells into nuclear and cytoplasmic fractions. The levels of pre-miR-155 expression in the total cellular, nuclear and cytoplasmic fractions were determined by real time PCR. The kinetics of pre-miR-155 expression in total cellular RNA (Figure 3A) mirrored that of pri-miR-155 (Figure 1A). Pre-miR-155 expression was induced quickly by LPS and peaked at 2 hours. IL-10 inhibition of pre-miR-155 was observed at 2 hours. The kinetic profiles of pre-miR-155 in nuclear and cytoplasmic RNA fractions were quite similar to that in total RNA, indicating that neither LPS nor IL-10 regulated or altered the nuclear export of pre-miR-155.

### IL-10 Inhibits LPS Induction of miR-155 via SHIP1 and STAT3

**Figure 4:**
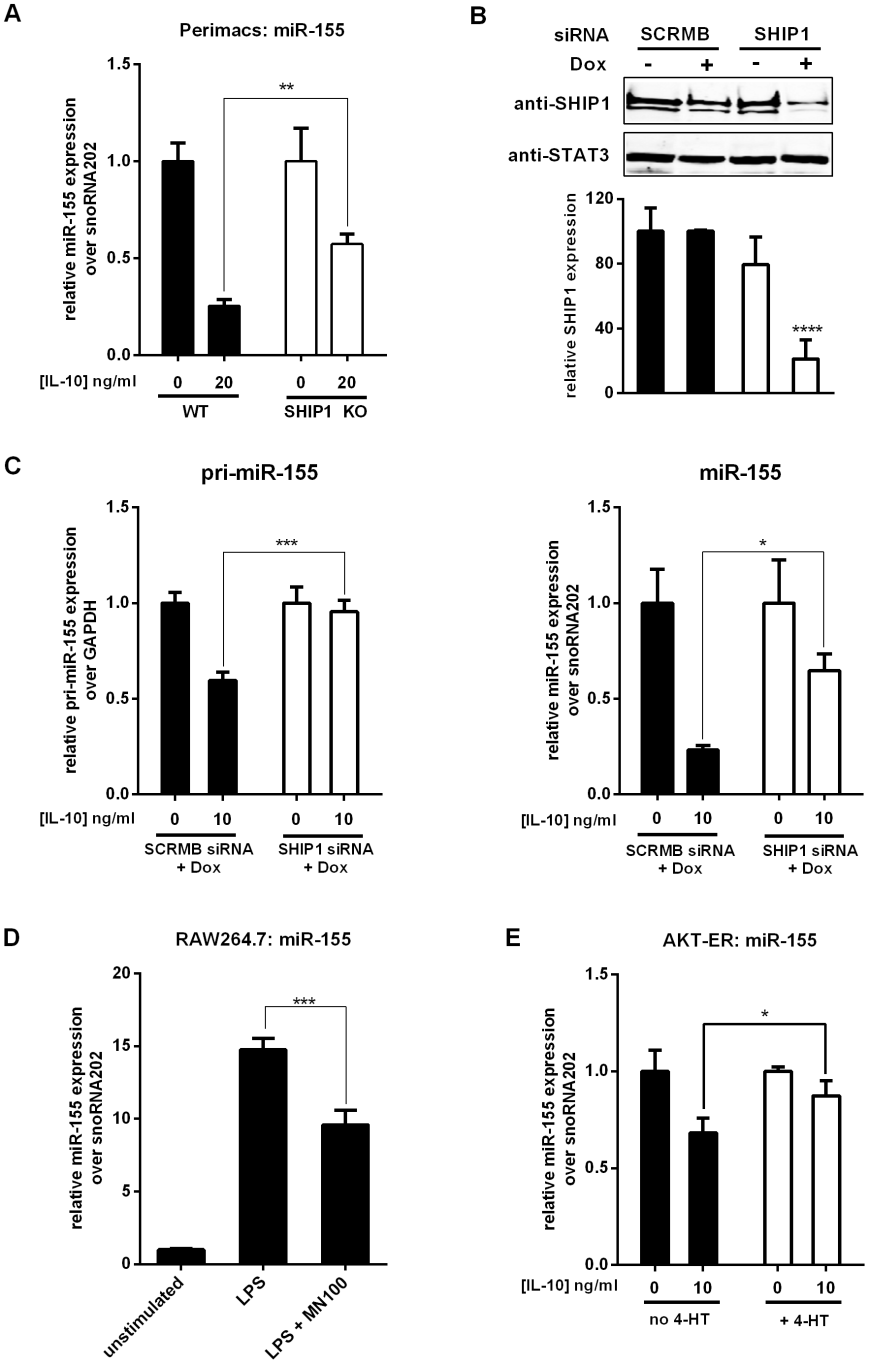
SHIP1 mediates IL-10 inhibition of miR-155. (A) Perimacs were extracted from either WT or SHIP1 KO mice, and stimulated with 10 ng/ml LPS or LPS +10 ng/ml IL-10. miR-155 expression levels were measured by real time PCR and plotted relative to the LPS alone sample in each cell type. (B) SCRMB and SHIP1 siRNA transduced cells were treated with 2 µg/ml Dox for 48 hours or left untreated prior to immunoblotting analysis for SHIP1 and STAT3 (loading control). Band intensities were quantified using the Quantity One Software. Statistical significance between treatments were calculated by a two-way ANOVA test with a 95% confidence (****p<0.0001). (C) SCRMB and SHIP1 siRNA transduced cells were treated with 2 µg/ml Dox for 48 hours and then stimulated with LPS +/− IL-10 for 2 or 4 hours. Expression levels of pri-miR-155 in the 2-hour samples and miR-155 in the 4-hour samples were measured by real time PCR and plotted relative to the LPS alone samples. (D) RAW264.7 cells were left untreated, treated with 10 µM AQX-MN100 or ethanol control for 30 minutes before being stimulated with LPS or LPS+AQX-MN100 for 4 hours. Expression level of miR-155 was measured by real time PCR and plotted relative to LPS samples. (E) AKT-ER transduced cells were treated with 150 nM 4-HT for 20 minutes or left untreated before being stimulated by LPS +/− IL-10 for 4 hours. Expression level of miR-155 was measured by real time PCR and plotted relative to the LPS alone samples. Statistical significance between stimulation conditions was calculated by a two-way ANOVA test with a 95% confidence (**p<0.01, ***, *p*<0.001, ****p<0.0001). Results were observed in at least two independent experiments.

McCoy *et al.* found that IL-10 inhibition of miR-155 expression required the presence of STAT3 protein [37]. The STAT3 pathway is the best characterized pathway downstream of the IL-10 receptor, however we recently found that IL-10 also signals through the phosphatase SHIP1 [15] (Ming-Lum *et al*., submitted), so we investigated the contribution of SHIP1 to IL-10 inhibition of pre-miR-155 and mature miR-155 levels. We tested the ability of IL-10 to inhibit miR-155 in perimacs from wild-type or SHIP1 knockout (SHIP1 KO) mice. As shown in Figure 4A, IL-10 could not inhibit miR-155 expression to the same extent in SHIP1 deficient cells as compared to wild-type cells.

To confirm these findings, we generated RAW264.7 cell lines in which SHIP1 protein levels were reduced by RNA silencing. siRNA sequence targeting SHIP1 or a scrambled (SCRMB) sequence was cloned into the pTRIPZ lentiviral vector which contains miRNA-like processing elements to express the siRNA sequence under the control of a doxycycline (Dox) regulated promoter. The addition of Dox to the SHIP1 siRNA transduced cells reduced SHIP1 protein expression by 80% (Figure 4B). Similar to that observed in wild-type perimacs, IL-10 inhibited pri-miR-155 and miR-155 in the SCRMB siRNA transduced cells (Figure 4C). Similar to SHIP KO perimacs, the SHIP1 siRNA transduced cells had reduced IL-10 inhibition of mature miR-155 (Figure 4C).

Our data suggested that SHIP1 negatively regulated LPS-induced miR-155 expression. To determine whether activation of SHIP1 alone could inhibit miR-155 expression, we made use of a small molecule SHIP1 activator, AQX-MN100, which binds to the allosteric activation site on SHIP1 and activates its phosphatase activity [44]. AQX-MN100 is specific for SHIP1 and does not activate even the closely related SHIP2 inositol phosphatase [44]. We found AQX-MN100 inhibited miR-155 expression in LPS-stimulated macrophages (Figure 4D), suggesting that SHIP1 activation alone can reduce miR-155 levels. Notably, SHIP1 activation alone does not reduce miR-155 levels to the same extent as IL-10 (Figure 4C), suggesting other IL-10 regulated signalling pathways contribute to IL-10’s effect.

Since SHIP1 is a negative regulator of the PI3K/AKT pathway [45], we reasoned that the PI3K/AKT pathway would have a positive role in miR-155 expression. We tested this hypothesis by expressing a conditionally active form of AKT in RAW264.7 cells. This AKT-Estrogen Receptor (ER) fusion protein is activated by the addition of 4-hydroxytamoxifen (4-HT), which displaces HSP90 and allows AKT-ER access to its substrates [46]. Pretreating the cells with 150 nM 4-HT for 20 minutes was sufficient to activate AKT-ER, indicated by the increased phosphorylation of GSK3, a substrate of AKT [47] (Figure S3). The untreated cells and the 4-HT treated cells produced similar miR-155 level in respond to LPS, but their responses to IL-10 differed (Figure 4E). IL-10 inhibition of miR-155 was impaired in 4-HT treated AKT-ER expressing cells (Figure 4E).

**Figure 5:**
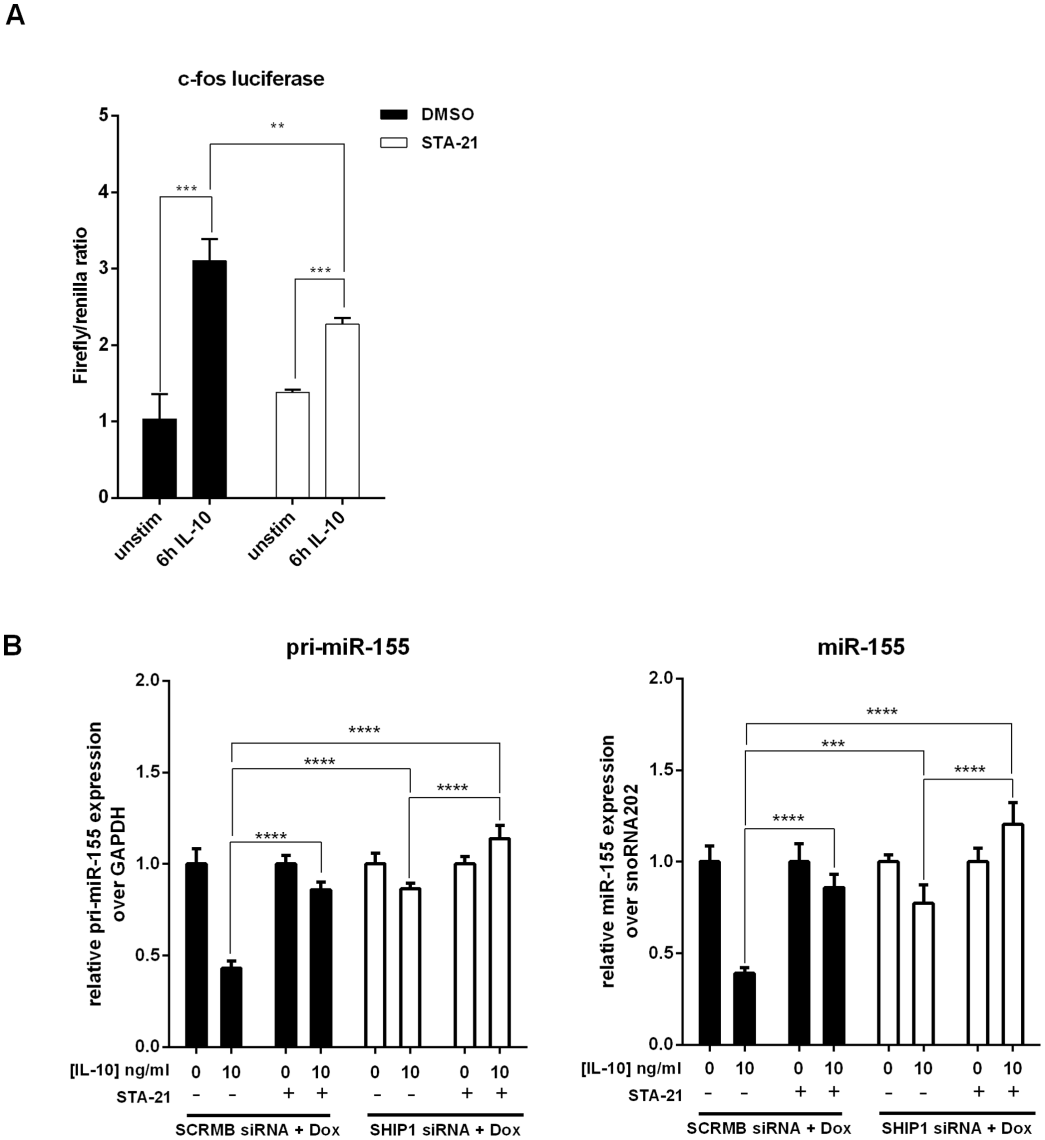
SHIP1 and STAT3 play additive roles in IL-10 inhibition of miR-155. (A) RAW264.7 cells were transfected with the c-fos promoter reporter and TK-Renilla, and were pretreated with DMSO or 30 µM STA-21 for 1 hour prior to IL-10 stimulation for 6 hours. Luciferase activity was measured and plotted as firefly/renilla ratio. (B) SCRMB and SHIP1 siRNA transduced cells were treated as Figure 4C except the cells were pretreated with DMSO or 30 µM STA-21 for 1 hour prior to stimulation. Expression levels of pri-miR-155 at 2 hours and miR-155 at 4 hours were measured by real time PCR and plotted relative to the LPS alone samples. Statistical significance between stimulation conditions was calculated by a two-way ANOVA test with a 95% confidence (**p<0.01, ***p<0.001, ****p<0.0001). Results were observed in at least two independent experiments.

We then examined whether the contribution of STAT3 and SHIP1 to IL-10 inhibition of mature miR-155 were additive or redundant. To do this we made use of the synthetic STAT3 inhibitor, STA-21 [48]. We first tested the efficacy of STA-21 by testing its ability to inhibit IL-10 activation of the STAT3-responsive, c-fos promoter luciferase reporter [15], [38]. RAW264.7 cells were transiently co-transfected with the c-fos firefly luciferase and SV40 renilla luciferase control constructs. Cells were then treated with STA-21 or vehicle control, and stimulated with IL-10 or left unstimulated. As shown in Figure 5A, IL-10 induced the activity of the c-fos promoter, but pretreatment of 30 µM STA-21 reduced this induction. We then added STA-21 to SCRMB or SHIP1 siRNA transduced cells and measured the levels of pri-miR-155 and mature miR-155. As shown in Figure 5B, STA-21 impaired IL-10’s ability to inhibit pri-miR-155 and mature miR-155 in both cells. In cells lacking SHIP1, the effect of STA-21 was more pronounced than untreated cells. In fact, the expression of pri-miR-155 and miR-155 was enhanced, rather than inhibited by IL-10. These data suggest SHIP1 and STAT3 play additive, non-redundant roles in IL-10 inhibition of miR-155.

## Discussion

miRNAs regulate both immune cell development and function [49]. In particular, miR-155 is extensively involved in different aspect of the immune system including haematopoiesis [50], T cell development [30], B cell differentiation [51], dendritic cell maturation [52], as well as mediating inflammation [25], [29]–[35]. Enhanced miR-155 expression is associated with various human diseases such as rheumatoid arthritis [53] and cancers [27]–[29]. The multiple roles of miR-155 are mediated by its numerous targets that include transcription factors, protein receptors, kinases and other signalling molecules [54]. Because of miR-155’s pro-inflammatory role in macrophage activation, we examined whether IL-10 regulated miR-155 levels in our cells and if so, whether SHIP1 played a role. We found that IL-10 was able to inhibit the expression of miR-155 in activated macrophages (Figure 1A–B), which is consistent with previous report [37]. However, unlike McCoy *et al.* [37], we found that neither LPS nor IL-10 regulated miR-155 at the transcriptional level.

miRNAs can be regulated at multiple steps: transcription, nuclear export and maturation [32]. We, Ruggiero *et al.* and McCoy *et al.* all observe upregulation of pri-miR-155 RNA. Although Ruggiero *et al.* and we both conclude that pri-miR-155 levels are regulated primarily through post-transcriptional mechanisms, McCoy *et al.* conclude that pri-miR-155 levels rise through increased transcription. Ruggiero *et al.* used chromatin immunoprecipitation and sequencing to show that pri-miR-155 transcription rates do not change with LPS stimulation. We came to the same conclusion using BIC promoter luciferase reporter assays and cycloheximide experiments. Our results with the BIC promoter reporter differed from McCoy *et al.*’s BIC promoter reporter assays in that they found LPS stimulated reporter activity while we did not. We do not know why our results differ, but we note that McCoy *et al.* based their conclusion solely on luciferase reporter experiments, without using any other additional experimental approaches such as the ones Ruggiero *et al.* and we used. Although neither LPS nor IL-10 altered BIC promoter activity, the level of pri-miR-155 transcript increased with LPS and decreased with IL-10 treatment. Steady state transcript levels is controlled not only by transcriptional activity, but also maintained through transcript stability. Therefore we used ActD and CHX treatments to examine whether pri-miR-155 transcript levels were being kept low in resting cells through degradation (Figure 2C–D). ActD reduced pri-miR-155 levels while CHX enhanced it, indicating that pri-miR-155 is constitutively transcribed and at the same time degraded in unstimulated cells.

Interestingly, although IL-10 significantly decreases the levels of pri-miR-155 by 2 hours after addition, significant decreases in mature miR-155 do not occur until 4 hours after stimulation. This discrepancy in kinetics suggests an additional layer of control past the regulation of pri-miR-155 levels. Thus, we examined whether IL-10 regulated the nuclear export of pre-miR-155 by fractioning total RNA into nuclear and cytoplasmic RNA. We found that the kinetics of pre-miR-155 expression in the nucleus and the cytoplasm were similar suggesting that nuclear export of miR-155 was not regulated by IL-10. From these observations, we deduced that IL-10 is likely regulating the processing of pre-miR-155 to functional, mature miR-155. Emerging evidence shows that miRNA biogenesis or processing can be regulated at the post-transcriptional steps by different RNA-binding proteins that modulate Drosha or Dicer activities [55]–[57]. In particular, the RNA binding protein KSRP was found to be required for miR-155 maturation in response to LPS stimulation in macrophages [24]. Furthermore, the ability of KSRP to support miRNA maturation was found to be stimulated by AKT-mediated phosphorylation [58]. Future studies in the lab are directed to examine whether IL-10 may modulate the function of KSRP to inhibit the production of mature miR-155.

IL-10 function is well known to be mediated by the transcription factor STAT3 [12]–[14], [59], [60] and STAT3 is involved in IL-10 inhibition of miR-155 [37]. We recently found that the phosphatase SHIP1 is also involved in mediating IL-10 inhibition of LPS-induced TNFα production and AKT activation [15] (Ming-Lum *et al.*, submitted). We now report that IL-10 inhibition of miR-155 expression was impaired in macrophages lacking SHIP1 but such inhibition could be achieved by the addition of the SHIP1 activator, AQX-MN100 (Figure 4). The addition of the STAT3 inhibitor STA-21 further reduced IL-10 inhibition (Figure 5). The additive effect of SHIP1 knockdown and STAT3 inhibition suggests that SHIP1 and STAT3 regulate miR-155 expression through independent mechanisms.

Consistent with the fact that SHIP1 is a negative regulator of the PI3K/AKT pathway [45], we found that 4-HT mediated activation of AKT-ER abolished IL-10 inhibition of miR-155 expression (Figure 4C), indicating a positive role of AKT in miR-155 expression. This finding appears to disagree with a previous study in which a myristylated, constitutively active AKT reduced LPS-induced miR-155 in macrophages [61]. The difference in conclusions between our and Androulidaki *et al.*’s studies may be due to the use of the constitutively active AKT in Androulidaki *et al.*’s study. Persistent activation of AKT may change the nature of the cells. In contrast, the AKT-ER fusion protein we used in our experiments is only active when we add 4-HT.

**Figure 6:**
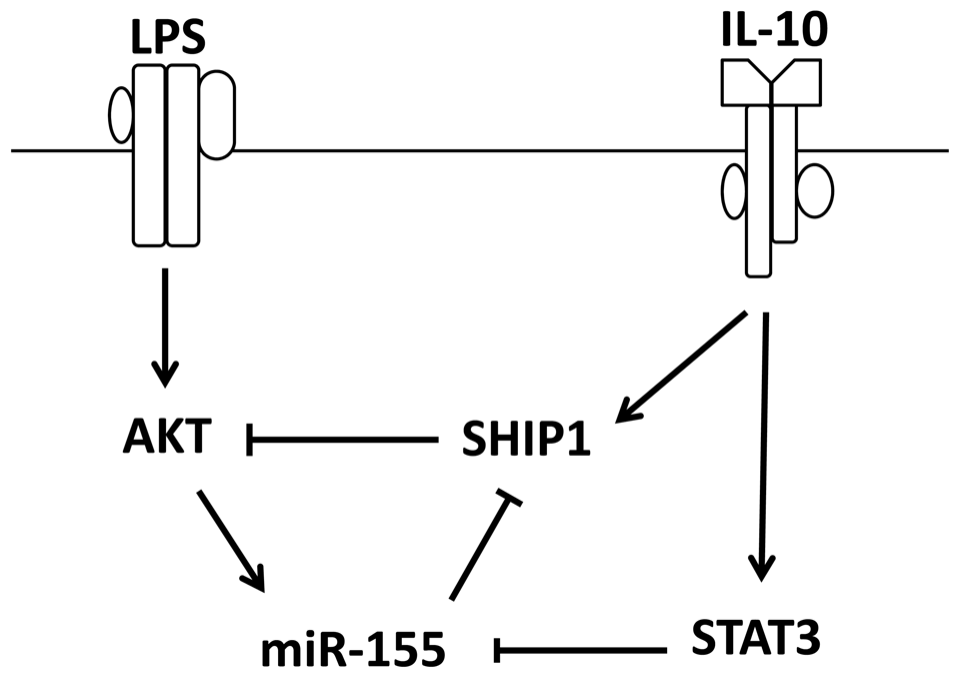
Schematic diagram of IL-10 inhibition of miR-155 expression via both SHIP1 and STAT3.

SHIP1 is a well-characterized miR-155 target [32], [33]. Thus, the involvement of SHIP1 in IL-10 inhibition of miR-155 expression constitutes an elegant regulatory circuit composed of SHIP1, AKT and miR-155 (Figure 6): LPS-induced activation of AKT promotes the expression of miR-155, which suppresses SHIP1, to allow PI3K/AKT pro-inflammatory events. On the other hand, IL-10 mediated activation of SHIP1 inhibits AKT signalling and reduces miR-155 expression. As a result, SHIP1 protein translation is resumed and further suppresses macrophage activation.

Together, our data supported a new mode of action for the anti-inflammatory cytokine IL-10 in which IL-10 controls the overall level of functional miR-155 by regulating the stability of pre-miR-155 and its maturation through SHIP1 and STAT3-dependent mechanisms.

## Supporting Information

Figure S1 **The BIC promoter reporter was unresponsive to LPS and IL-10.** The BIC promoter reporter constructs were transfected into RAW264.7 cells using either XtremeGene HP transfection reagent or GeneJuice transfection reagent according to manufacturer’s instruction. Cells were then rested in medium containing 9% or 5% serum for 24–48 hours before stimulation for the indicated time. Luciferase activity was measure with Dual-Glo Luciferase Assay System, and plotted as % of the unstimulated samples.
(TIF)

Figure S2 **CHX treatment altered the expression level of GAPDH and actin, but not that of 18S rRNA.** RAW264.7 cells were treated with DMSO, CHX, ActD, LPS or LPS+CHX for 1 or 2 hours prior to RNA extraction and determination of pri-miR-155, GAPDH, actin and 18S rRNA levels by real time PCR. Raw Ct values of pri-miR-155 were plotted against those of each normalization control.
(TIF)

Figure S3 **4-HT treatment in the AKT-ER cells increased phosphorylation of G3K3.** AKT-ER cells were either untreated or treated with 150 nM 4-HT for 20 minutes or 2 hours prior to immunoblotting analysis for phospho-GSK3 (pGSK3) and vinculin (loading control).
(TIF)
