# Supplementary figures and images for: Interleukin-10 Inhibits Lipopolysaccharide Induced miR-155 Precursor Stability and Maturation
Source: PLoS One. 2013 Aug 12;8(8):e71336. doi: 10.1371/journal.pone.0071336 (PMC3741136; doi:10.1371/journal.pone.0071336)

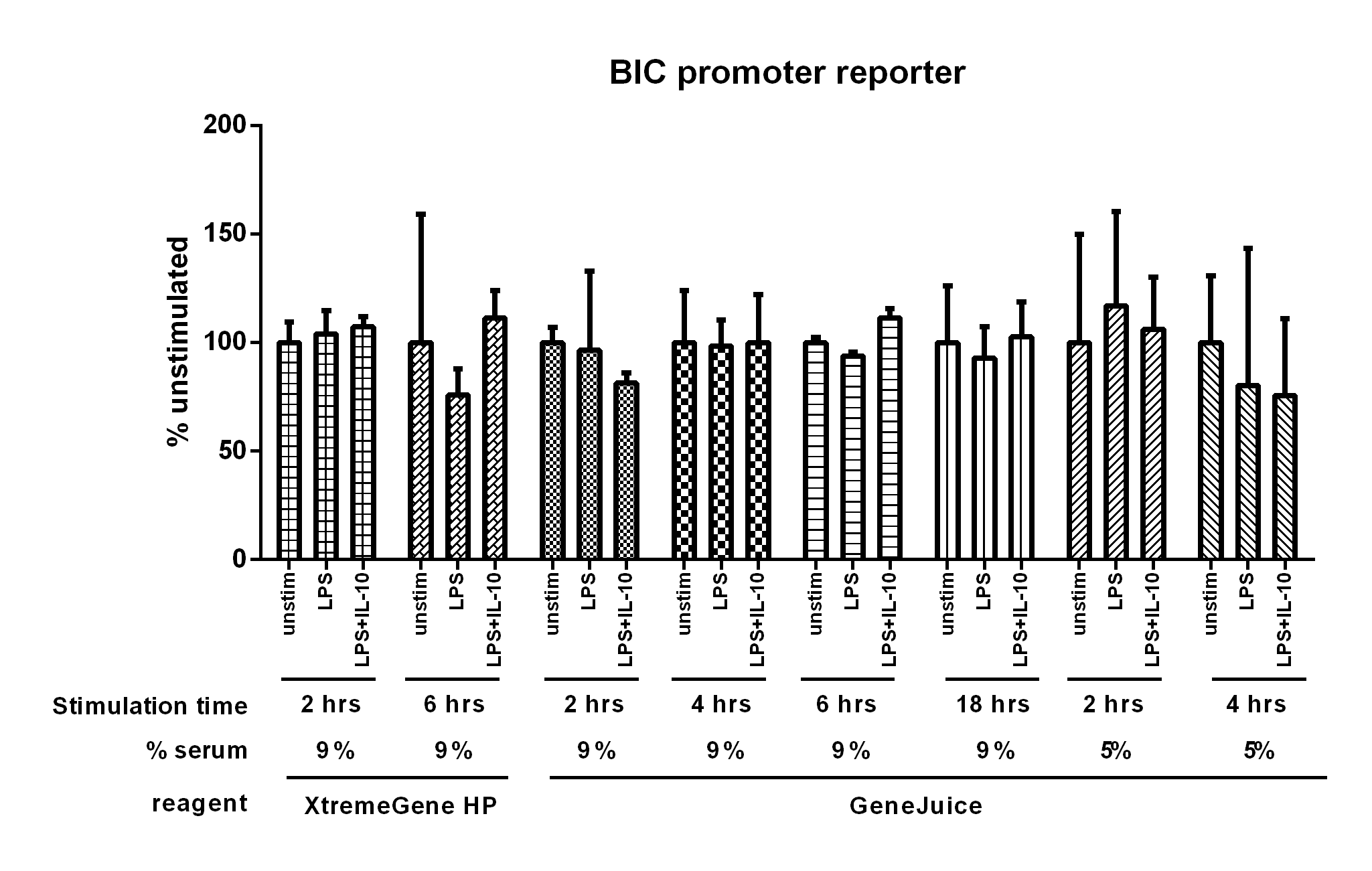

Supplement: Figure S1 — The BIC promoter reporter was unresponsive to LPS and IL-10. The BIC promoter reporter constructs were transfected into RAW264.7 cells using either XtremeGene HP transfection reagent or GeneJuice transfection reagent according to manufacturer’s instruction. Cells were then rested in medium containing 9% or 5% serum for 24–48 hours before stimulation for the indicated time. Luciferase activity was measure with Dual-Glo Luciferase Assay System, and plotted as % of the unstimulated samples. (TIF) [file pone.0071336.s001.tif]

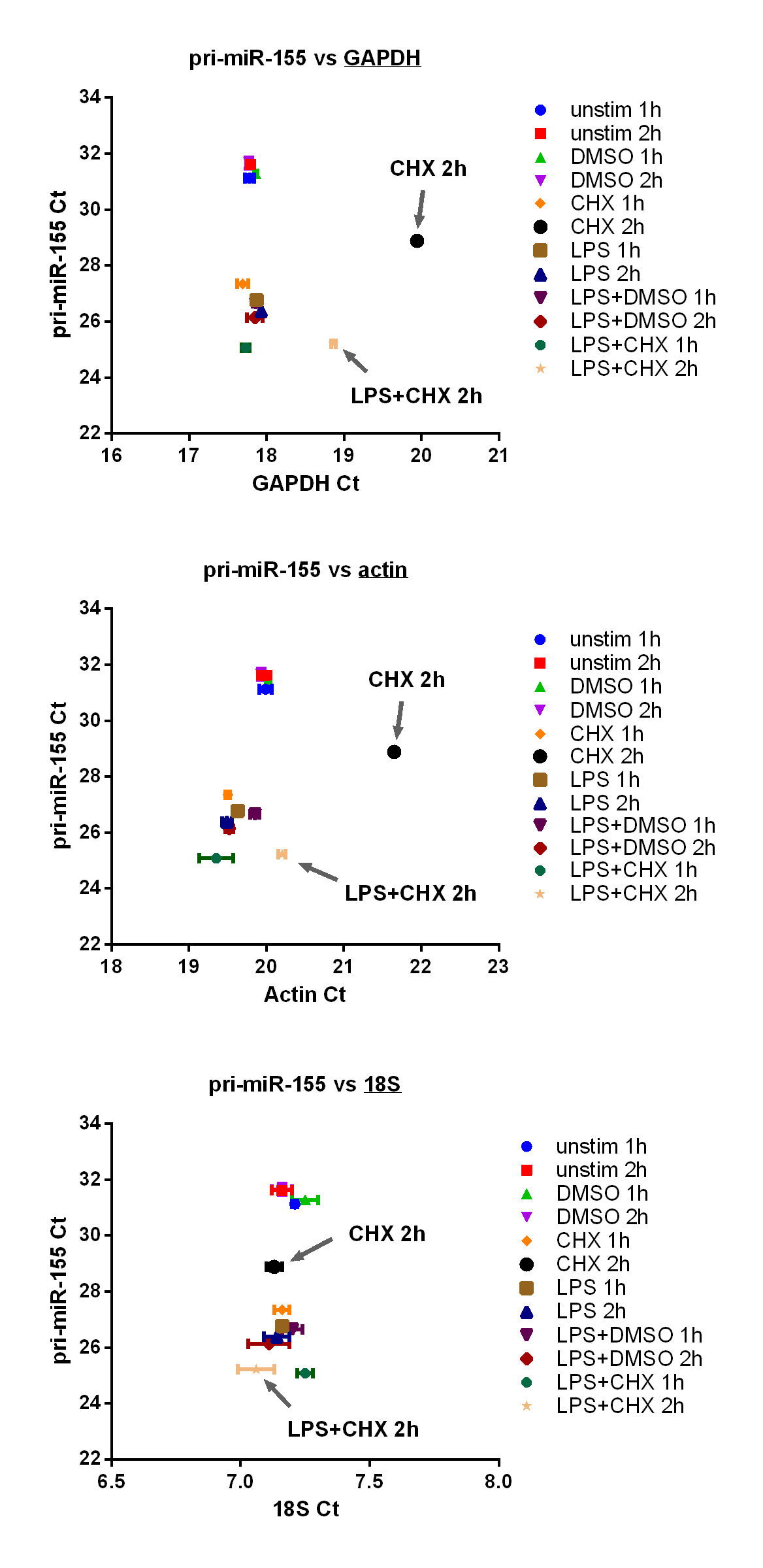

Supplement: Figure S2 — CHX treatment altered the expression level of GAPDH and actin, but not that of 18S rRNA. RAW264.7 cells were treated with DMSO, CHX, ActD, LPS or LPS+CHX for 1 or 2 hours prior to RNA extraction and determination of pri-miR-155, GAPDH, actin and 18S rRNA levels by real time PCR. Raw Ct values of pri-miR-155 were plotted against those of each normalization control. (TIF) [file pone.0071336.s002.tif]

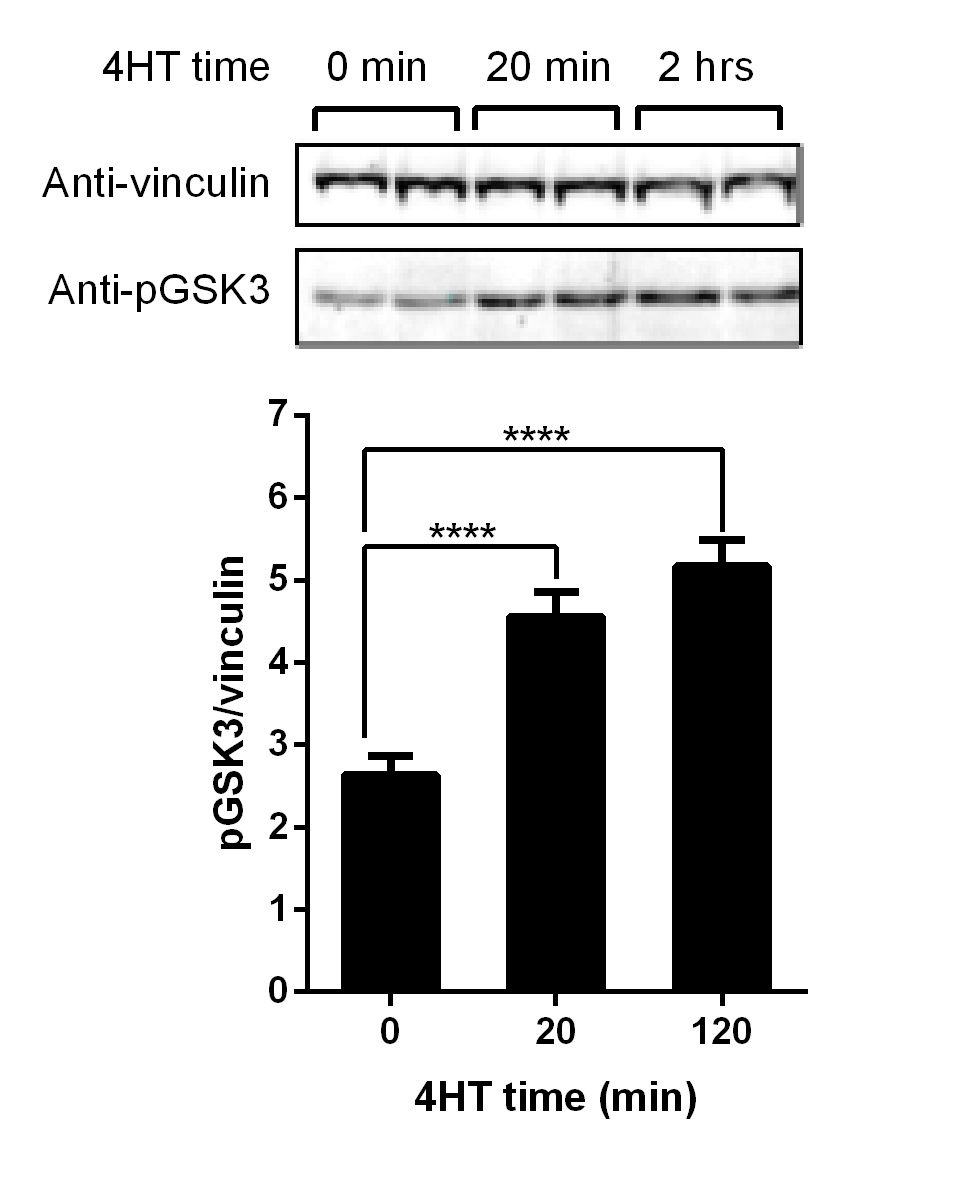

Supplement: Figure S3 — 4-HT treatment in the AKT-ER cells increased phosphorylation of G3K3. AKT-ER cells were either untreated or treated with 150 nM 4-HT for 20 minutes or 2 hours prior to immunoblotting analysis for phospho-GSK3 (pGSK3) and vinculin (loading control). (TIF) [file pone.0071336.s003.tif]
